# Mechanisms and Critical Thresholds of Cold Storage Duration-Modulated Postharvest Quality Deterioration in Litchi Fruit During Ambient Shelf Life

**DOI:** 10.3390/foods15010176

**Published:** 2026-01-05

**Authors:** Hai Liu, Zhili Xu, Longlong Song, Lilang Li, Yan Liao, Hui Du, Fengjun Li

**Affiliations:** 1 Guangdong Provincial Key Laboratory of Aquatic Product Processing and Safety, Guangdong Provincial Science and Technology Innovation Center for Subtropical Fruit and Vegetable Processing, Guangdong Province Engineering Laboratory for Marine Biological Products, Key Laboratory of Advanced Processing of Aquatic Product of Guangdong Higher Education Institution, College of Food Science and Technology, Guangdong Ocean University, Zhanjiang 524088, China; liuhai1187@163.com (H.L.); 13531086529@163.com (Z.X.); songll0007@126.com (L.S.); langll@gdou.edu.cn (L.L.); liaoyan39@163.com (Y.L.); wdh2000@139.com (H.D.); 2 Collaborative Innovation Center of Seafood Deep Processing, Dalian Polytechnic University, Dalian 116034, China

**Keywords:** litchi, cold storage duration, browning, antioxidant capacity, lipid peroxidation, quality

## Abstract

While cold storage is essential to extend the postharvest preservation of litchi fruit, the abrupt transfer to ambient temperature during supply chain transitions may trigger rapid quality degradation. However, the comprehensive mechanisms and critical threshold of post-transfer quality deterioration remain insufficiently characterized. In this study, litchi fruits were stored at 4 °C for 10, 20, and 30 days, followed by simulated shelf life at 25 °C. Key indicators, including appearance quality, antioxidant capacity, lipid peroxidation, and enzymatic oxidation, were monitored, and principal component analysis (PCA) was used to determine quality deterioration thresholds. Litchi subjected to 30 d of cold storage exhibited significantly accelerated pericarp browning compared to those stored for 20 d and 10 d, with the browning index increasing by 25.7% (vs. 20 d) and 41.9% (vs. 10 d), respectively, after 24 h of ambient exposure. This was accompanied by a significant impairment of the antioxidant system. Compared to the fruits stored for 10 d and 20 d, the activities of key antioxidant enzymes (SOD, CAT, and APX) were substantially decreased in the 30 d group, with reductions ranging from approximately 9% to 28%. Concurrently, the non-enzymatic antioxidant capacity also declined. Meanwhile, 30 d of storage activated the browning-related enzymes: anthocyanase and peroxidase (POD) activities increased by 1.2- to 3.6-fold, and poly-phenol oxidase (PPO) activity increased by 11% to 37%, compared to the 10 d and 20 d groups, respectively. In contrast, phenylalanine ammonia lyase (PAL) activity was inhibited by 56.9%. It also enhanced membrane lipid metabolism disorders, which aggravated cell structure damage and oxidative stress. For practical application, PCA identified 10 d (4 °C) + 6 h (25 °C), and 20 d (4 °C) + 12 h (25 °C) as the optimal and critical quality thresholds, respectively. This study reveals the interactive regulatory relationship between cold storage duration and ambient exposure time mediated by oxidative stress, enzymatic browning, and membrane lipid metabolism, providing a theoretical basis for developing time-temperature-quality models to reduce postharvest losses in litchi.

## 1. Introduction

As a non-climacteric subtropical to tropical fruit, litchi (*Litchi chinensis* Sonn.) has a high economic value for its vivid red pericarp and delicious, juicy, and crisp aril [1]. However, litchi fruit is highly perishable after harvest due to pericarp browning, which can lose up to 70% of commercial value within 48 h at room temperature [2]. Therefore, developing effective preservation technologies and elucidating the mechanisms underlying quality deterioration are critical for the sustainable development of the litchi industry.

Low-temperature storage (0–5 °C) has become the dominant postharvest preservation technology for litchi, as it can effectively extend the storage period to 1–4 weeks [3]. However, in the actual supply chain, during the processes of cold storage outbound, transportation, and terminal sales, the fruit inevitably experiences abrupt temperature fluctuations from low to ambient temperatures [4]. Such sudden temperature changes often disrupt the low-temperature acclimation balance established by the fruit, triggering a series of physiological and metabolic disorders [5,6]. Although the precise mechanisms of this phenomenon are complex, it primarily involves two interconnected processes: rapid pericarp browning, driven by the activation of enzymes such as polyphenol oxidase (PPO) and peroxidase (POD) [7,8,9], and severe oxidative stress, resulting from the collapse of antioxidant systems [10]. Furthermore, dysregulation of membrane lipid metabolism has been recognized as an early event under both low-temperature stress and temperature fluctuation injury [11,12,13].

This post-cooling accelerated deterioration often causes more severe damage than continuous low-temperature stress alone, and has attracted increasing research attention in recent years [3,4,5,6]. However, despite these insights, two critical research gaps remain. First, most existing studies have focused on quality changes during shelf life after a single, fixed period of cold storage (e.g., 15 or 20 days) [3,4,5,6]. However, a systematic understanding of the quality deterioration pattern under the dual-factor interaction of cold storage duration and ambient exposure time has not yet been formed. Second, the critical threshold of cold storage duration, i.e., the duration beyond which litchi will undergo irreversible quality deterioration at ambient temperature, has not been clarified. Identifying this threshold is essential for guiding temperature management at key nodes throughout the supply chain.

To address these questions, a multi-temporal experiment with 10, 20, and 30 d cold storage periods (4 °C) followed by a 25 °C shelf-life simulation was designed. The aim of this research was to investigate cold duration effects on visual quality, antioxidant capacity, lipid peroxidation, and enzymatic oxidation of litchi fruit during ambient shelf time. Additionally, quality deterioration critical thresholds were identified through principal component analysis (PCA). This study elucidates the interaction between cold storage duration and ambient exposure time by integrating the analysis of physiological indicators involved in oxidative stress, enzymatic browning, and membrane lipid metabolism. This approach breaks away from the constraints inherent in research based on a single cold storage cycle. Moreover, the foundation for developing time-temperature-quality models to reduce postharvest losses and enhance the commercial viability of litchi supply chains can be established.

## 2. Materials and Methods

### 2.1. Plant Materials and Treatments

Litchi fruits (cv. Huaizhi) at 80% maturity (characterized by 80% red pericarp coverage, average weight of 25.5 ± 0.5 g, and pulp soluble solid content of 19.1 ± 0.8%) were harvested from a commercial orchard in Zhanjiang, China. The fruits exhibiting uniform shape and color without blemishes or disease symptoms were selected for the experiment. A total of 1350 fruits were randomly divided into three groups. Fruit treatment followed the protocol described in our previous study [3]. Briefly, the fruits were immersed in 0.1% thiabendazole solution for 3 min, a treatment duration and concentration established in prior work to suppress microbial growth without compromising postharvest quality [3]. The fruits were then air-dried at 25 °C for 1 h. Subsequently, the treated fruits were packaged in 0.03 mm-thick polyethylene bags (30 fruits per bag). Each bag was pre-punched with 4 small holes (2 mm diameter) to ensure adequate ventilation, and no moisture absorbers or atmosphere stabilizers were added inside the bags. The packaged fruits were stored at 4 °C for 10, 20, or 30 d. Upon completion of each storage period, the bagged fruits were transferred to shelf-life conditions (25 °C, 80–90% relative humidity) for 24 h. During the shelf-life period, samples were collected at 6 h intervals, and the browning index was immediately evaluated.

### 2.2. Assessment of Pericarp Browning Index and Moisture Content

The browning index was evaluated as described in our previous study [3], with 3 independent biological replicates (each replicate consisting of 30 individual fruits).

To determine the moisture content, 10 g of litchi pericarp was obtained and oven-dried at 80 °C until constant weight (72 h). The sample was then allowed to cool in a desiccator prior to final weighing, and the moisture content was calculated according to the following formula:

water content (%) = (FW − DW)/FW.

where FW = fresh weight of pericarp, DW = dry weight of pericarp.

### 2.3. Determinations of Content of Anthocyanins, Total Phenolics, Hydrogen Peroxide (H_2_O_2_), and Malondialdehyde (MDA)

One gram of litchi pericarp was crushed with liquid nitrogen and added to a 1% HCl-methanol solution. The mixture was then extracted at 25 °C for 2 h and subsequently filtered to obtain the filtrates for the measurement of total phenolics. The total phenolic content was assayed based on the Folin–Ciocalteu method [14], and the pH-differential method was used to determine anthocyanin content [15]. A quantity of 10 g of pericarp from 30 litchis was ground, and 1% HCl was added for extraction until the pericarp was colorless. The extracts were filtered, and the filtrate was diluted with 0.4 M KCl-HCl buffer (pH 1.0) and citrate-phosphate (citric acid/Na_2_HPO_4_, pH 4.5), respectively. Thereafter, the absorbance at 510 nm was measured.

H_2_O_2_ content was determined using the titanium (IV) method [16]. Briefly, following dissolution of the sediment in 3 mL of 1 M H_2_SO_4_ (25 °C, 5 min), H_2_O_2_ concentration (in mmol kg^−1^ fresh weight (FW)) was determined by measuring the absorbance at 410 nm of the resulting solution and calculating against an external H_2_O_2_ standard. For malondialdehyde (MDA) measurement, a homogenate of 5 g pericarp (from 10 fruits) in 20 mL of 10% trichloroacetic acid was prepared, centrifuged (5000× *g*, 10 min), and the supernatant collected. Following the thiobarbituric acid (TBA) method [8], the supernatant was reacted with TBA reagent by heating in boiling water for 20 min, cooled, and assessed spectrophotometrically at 450, 532, and 600 nm. MDA content (µmol kg^−1^) was calculated as

[6.45 × (A_532_ − A_600_)] − [0.56 × A_450_].

### 2.4. Determinations of Superoxide Dismutase (SOD), Catalase (CAT), and Ascorbate Peroxidase (APX) Activities

For enzyme extraction, 5 g of pericarp from 10 litchi fruits were ground and homogenized in 20 mL of 50 mM potassium phosphate buffer (pH 7.0, containing 0.4 g PVP) on ice. After centrifugation at 20,000× *g* for 15 min at 4 °C, the supernatant was assayed for three key antioxidant enzymes according to Duan et al. [8].

SOD activity was quantified via the inhibition of NBT photoreduction, with one unit causing 50% inhibition.

CAT activity was determined by the consumption of H_2_O_2_ in a 3 mL system (0.5 mL extract, 0.5 mL 40 mM H_2_O_2_, 2 mL 50 mM phosphate buffer, pH 7.8), where a unit decreased A_240_ by 0.01 per minute.

APX activity was gauged by ascorbate oxidation at 290 nm, with a unit defined as oxidizing 1 μmol ascorbate per minute.

### 2.5. Evaluations of Reducing Power and Free Radical Scavenging Activity

Five grams of litchi pericarp were powdered in liquid nitrogen, followed by homogenization in 30 mL of methanol (containing 0.2 g sodium metabisulfite) while stirring at 25 °C for 30 min. Two layers of miracloth were used to filter the homogenate, and the residue was then extracted twice. Supernatants for antioxidant assays were processed by centrifugation (15,000× *g*, 15 min, 4 °C) and subsequently maintained at −80 °C until measurement.

The DPPH scavenging activity was evaluated according to the method of Pang et al. [17], with slight modification. A volume of 0.1 mL of sample extract was mixed with 2.9 mL of 0.1 mM DPPH-methanol solution. After 30 min of incubation in the dark at 25 °C, the absorbance at 517 nm was measured. The DPPH scavenging activity was expressed as a percentage (%) and estimated using the equation below:

DPPH scavenging activity (%) = (1 − [A − B]/A_0_) × 100,

where A, B, and A_0_ represent the absorbance of the sample, sample blank, and DPPH without the sample, respectively (*n* = 3).

The superoxide (O_2_^·−^) scavenging activity was determined as described [18]. The O_2_^·−^ were generated via photoinduction using a cool white fluorescent lamp (light intensity: 4000 lx). A volume of 0.25 mL of sample extract was added to 3 mL of reaction buffer solution, which contained 0.05 M phosphate buffer (pH 7.8), 63 μM NBT, 13 mM methionine, 100 μM EDTA, and 1.3 μM riboflavin. The absorbance of the reaction system was measured at 560 nm after 15 min of illumination at 25 °C.

The hydroxyl radical (·OH) scavenging activity (both site- and non-site-specific) was evaluated based on the inhibition of deoxyribose degradation [8], a method that reflects competition between antioxidants and either ·OH or iron ions.

The reducing power assay was measured and calculated following the published method, as described by Huang et al. [1].

### 2.6. Determination of Activities of PPO, POD, PAL, and Anthocyanase

The enzyme activities of PPO and POD were assayed using the published approach with minor modifications [19]. After adding 1 g of litchi pericarp to 0.2 M phosphate buffer (pH 6.8, including 1% PVP), the mixture was homogenized for 10 min at 4 °C. The activities of PPO and POD were measured using the supernatant obtained after a 15 min centrifugation at 15,000× *g* and 4 °C. Enzyme units were defined based on the change in absorbance per minute. One unit of PPO activity corresponded to an increase of 0.001 at 398 nm, while for POD, one unit equaled an increase of 0.1 at 470 nm.

PAL activity was assayed per Xu et al. [20], where a unit was regarded as the enzyme quantity inducing an absorbance increase of 0.01 at 290 nm per hour.

Determination of anthocyanase activity was conducted following the previous method [21]. The rate at which 1 nM of cyanidin-3-glucoside was broken down per minute at 40 °C was considered one unit of anthocyanase.

### 2.7. Determination of Phosphatidylcholine (PC), Phosphatidylinositol (PI), and Phosphatidic Acid (PA) Content

The extraction and content determination of the phospholipids were conducted following the method of Zhang et al. [22], with slight modifications. One gram of litchi pericarp was pulverized in liquid nitrogen and extracted with 15 mL of Folch reagent under ultrasonication for 1 h. After that, the samples were centrifuged at 9000× *g* and 4 °C for 20 min. The lower chloroform phase was gathered, then 1 mL of acetone was added, followed by vortex oscillation for 2 min. The samples were then dried with nitrogen and redissolved in 1 mL of Folch reagent, which was subsequently filtered through a filter membrane (0.22 μm) before HPLC analysis. A HPLC system equipped with a SIL100A column (250 mm × 4.8 mm, 5 μm) and an ELSD-LTII evaporative light-scattering detector was employed for the determination of the PC, PI, and PA content. The operating conditions included column temperature of 40 °C, wavelength of 254 nm, injection volume of 10 μL, and flow rate of 0.8 mL min^−1^. An isocratic elution was performed using a mixture of mobile phase A and mobile phase B (1:1, *v*/*v*). Mobile phase A consisted of triethylamine/acetic acid/isopropanol/n-hexane (0.8:15:170:814.2, *v*/*v*/*v*/*v*), and mobile phase B consisted of triethylamine/acetic acid/water/isopropanol (0.8:15:140:844.2, *v*/*v*/*v*/*v*). The parameters of the evaporative light scattering detector were set at 40 °C and 350 kPa. The retention times of the authentic standards were determined: PI, 21.8 min; PC, 25.0 min; PA, 29.9 min. For method validation, the calibration curves for all analytes showed excellent linearity (R^2^ > 0.998) across their respective concentration ranges, and the average recovery rates were between 95.2% and 97.5%, validating the method for quantitative phospholipid analysis in litchi pericarp. Quantification was achieved by comparison with the standard mixture of PC, PI, and PA.

### 2.8. Statistical Analysis

The study followed a completely randomized design with three replicates. Data are reported as mean ± standard errors (SE) and were analyzed in SPSS 25.0. Additionally, PCA was conducted within SPSS, and the corresponding graphs were generated using Origin 2021.

## 3. Results

### 3.1. Pericarp Browning, MDA Content, H_2_O_2_ Content, O_2_^·−^ Production Rate

**Figure 1:**
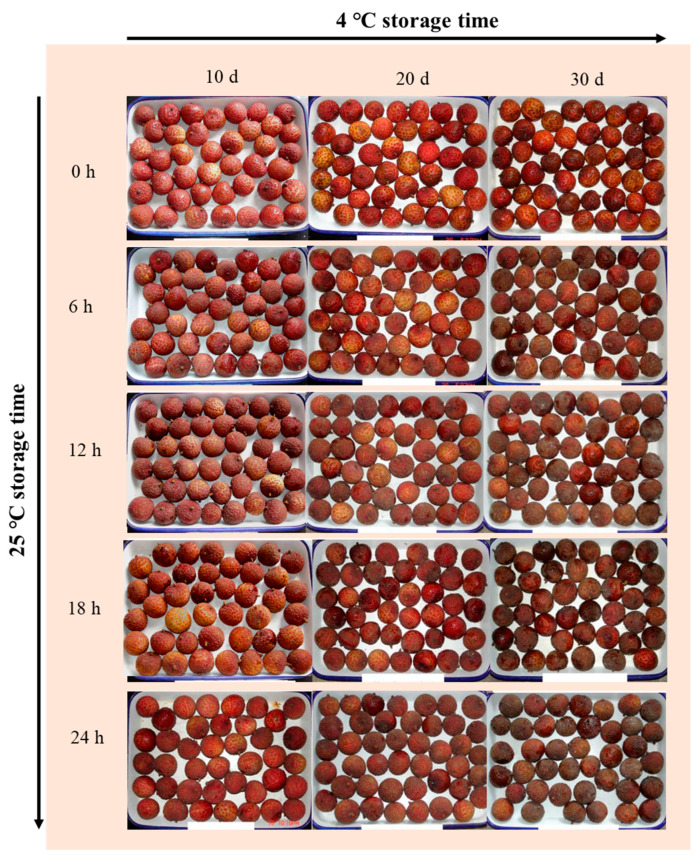
Appearance of litchi fruits stored for 10, 20, and 30 days at 4 °C during subsequent 24 h shelf time at 25 °C.

**Figure 2:**
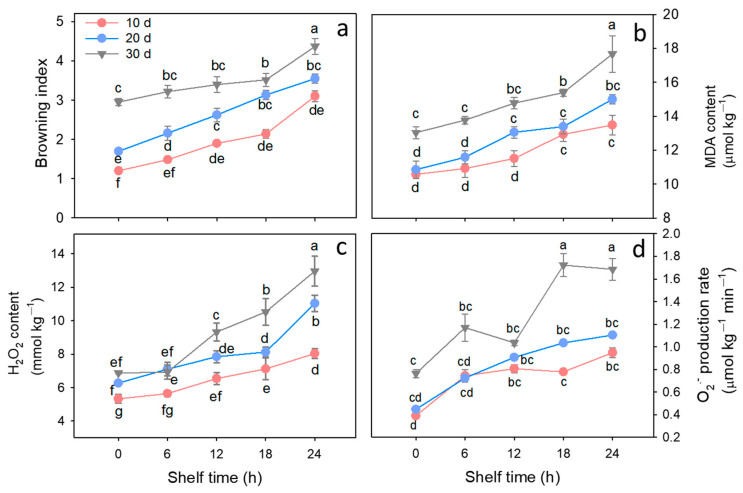
Changes in browning index (**a**), MDA content (**b**), superoxide production rate (**c**), and H_2_O_2_ content (**d**) of litchi fruits stored for 10, 20, and 30 days at 4 °C during the subsequent 24 h shelf time at 25 °C. Data are shown as means ± SE (*n* = 3). Lowercase letters denote statistically significant differences at *p* < 0.05.

The raw materials used in this experiment were standardized across all treatment groups. Figure S1 presented the litchi fruits harvested from the commercial orchard (Figure S1a) and their uniform status at 0 d (before cold storage, Figure S1b), confirming that the same batch of fruits was employed for all cold storage treatments (10/20/30 d at 4 °C). Regarding the post-storage quality changes, as showed in Figure 1, almost no or slight pericarp browning was observed in litchi fruits stored at 4 °C for 10 or 20 d. However, pericarp browning of cold-stored fruits for 30 d was obvious. After removal from low temperature, pericarp browning in litchi fruit exhibited a sharp increase as shelf time extended, and the extension of cold storage accelerated this process. In agreement with the change in visual appearance, fruits stored at 4 °C for 30 d had a browning index of 4.4 after 24 h of ambient storage, representing increases of 41.9% and 25.7% compared to those stored for 10 d and 20 d, respectively (Figure 2a). Consistent with the browning trend, pericarp water content gradually decreased during 4 °C cold storage and further declined under ambient conditions (Supplementary Table S1). Key oxidative stress markers were evaluated; MDA content in fruits subjected to 4 °C for 30 d was 13 μM kg^−1^ and significantly higher (*p* < 0.05) than those of fruits cold stored for 10 and 20 d (Figure 2b). Similar to the browning index, MDA content in fruits with 30 d of storage was higher than that in fruits with 10 or 20 d of storage throughout shelf time.

As shown in Figure 2c, the H_2_O_2_ content in fruits with 30 d of storage increased sharply during late shelf life, while those in fruits held for 10 and 20 d slowly increased throughout shelf life, except for an apparent increase in fruits held for 20 d (Figure 2c). Similarly, after 24 h of shelf time, the O_2_^·−^ production rates in litchi fruits stored at 4 °C for 10, 20, and 30 d were 0.38, 0.44, and 0.76 μM kg^−1^ min^−1^ (Figure 2d), respectively. Upon removal from low temperature, O_2_^·−^ production rate in fruits held for 30 d increased rapidly and reached 1.69 μM kg^−1^ min^−1^, while O_2_^·−^ production rates in fruits held for 10 or 20 d were 0.94 and 1.10 μM kg^−1^ min^−1^ after 24 h of ambient storage.

### 3.2. Antioxidant Enzyme Activities, Reducing Power, and Free Radical Scavenging Activity

**Figure 3:**
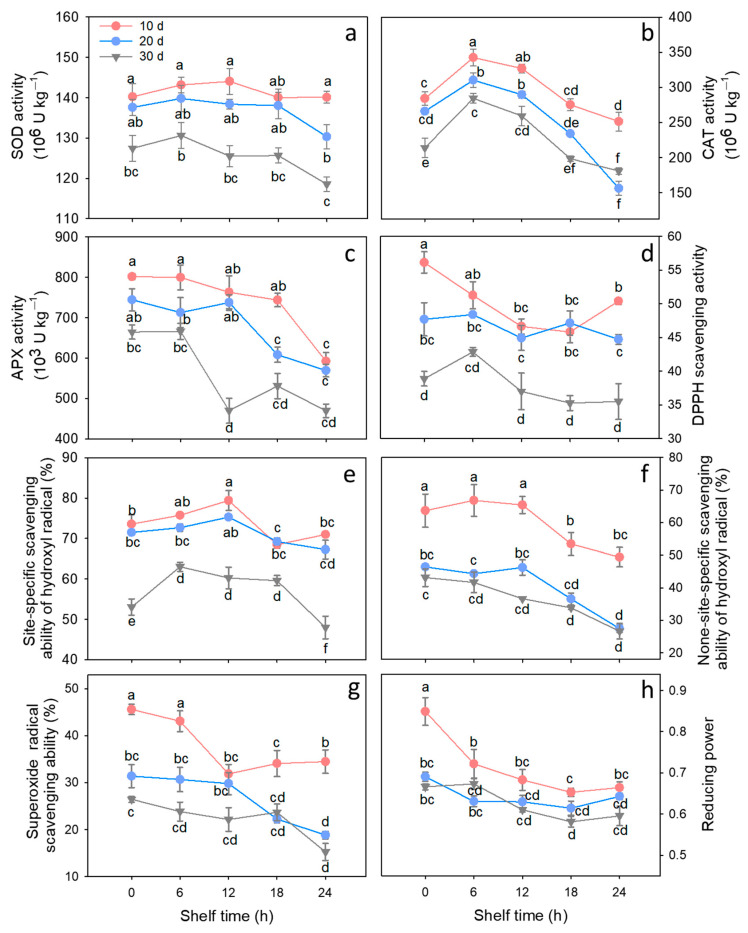
Changes in the activities of SOD (**a**), CAT (**b**), APX (**c**), scavenging capacity of DPPH radical (**d**), hydroxyl radical (site-specific (**e**) and non-site-specific (**f**)), superoxide anion (**g**), and reducing power (**h**) of litchi fruits stored for 10, 20, and 30 days at 4 °C during subsequent 24 h shelf time at 25 °C. Data are shown as means ± SE (*n* = 3). Lowercase letters denote statistically significant differences at *p* < 0.05.

Activity of SOD, CAT, and APX in litchi fruits stored for 30 d was lower than those in litchi fruits held for 10 and 20 d at 4 °C. Upon removal from low temperature, SOD activity in fruit stored at 4 °C for 10 d maintained at a high level, and the activity in fruits held for 20 d decreased only after 24 h of ambient storage. In comparison, the SOD activity in fruits held for 30 d tended to decrease, with levels being significantly lower (*p* < 0.05) than those in fruits held for 10 and 20 d throughout shelf time (Figure 3a). For CAT, the activity increased over the initial 6 h of shelf time, and then decreased markedly. Fruits stored at 4 °C for 30 d exhibited lower CAT activity in comparison to fruits held for 10 and 20 d during the subsequent shelf time (Figure 3b). APX activity in fruits held for 30 d decreased drastically, which was much lower (*p* < 0.05) compared to fruits held for 10 and 20 d throughout shelf time (Figure 3c).

As shown in Figure 3f, the non-site-specific ·OH scavenging activity in litchi fruits decreased as cold storage time and shelf time progressed. During the subsequent shelf time, the non-site-specific ·OH scavenging activity in fruits stored at 4 °C for 10 d was much higher than that in fruits with storage of 20 and 30 d. Concurrently, the site-specific activity displayed a distinct pattern, characterized by a slight initial rise followed by a decrease in cold-stored fruits. Moreover, the site-specific ·OH scavenging activity in fruits stored at 4 °C for 30 d was much lower (*p* < 0.05) than in fruits stored for 10 and 20 d throughout shelf life (Figure 3e). Similarly, the scavenging activity against DPPH and O_2_^·−^, and reducing power in litchi fruits decreased as the storage time extended (Figure 3d,g,h). After removal from low temperature, scavenging activity against DPPH and O_2_^·−^, and reducing power tended to decrease. Moreover, fruits that were held at 4 °C for 30 d had lower levels of reducing power and free radical scavenging activity than fruits that were stored for 10 and 20 d throughout shelf time.

### 3.3. Contents of PC, PI, and PA

**Figure 4:**
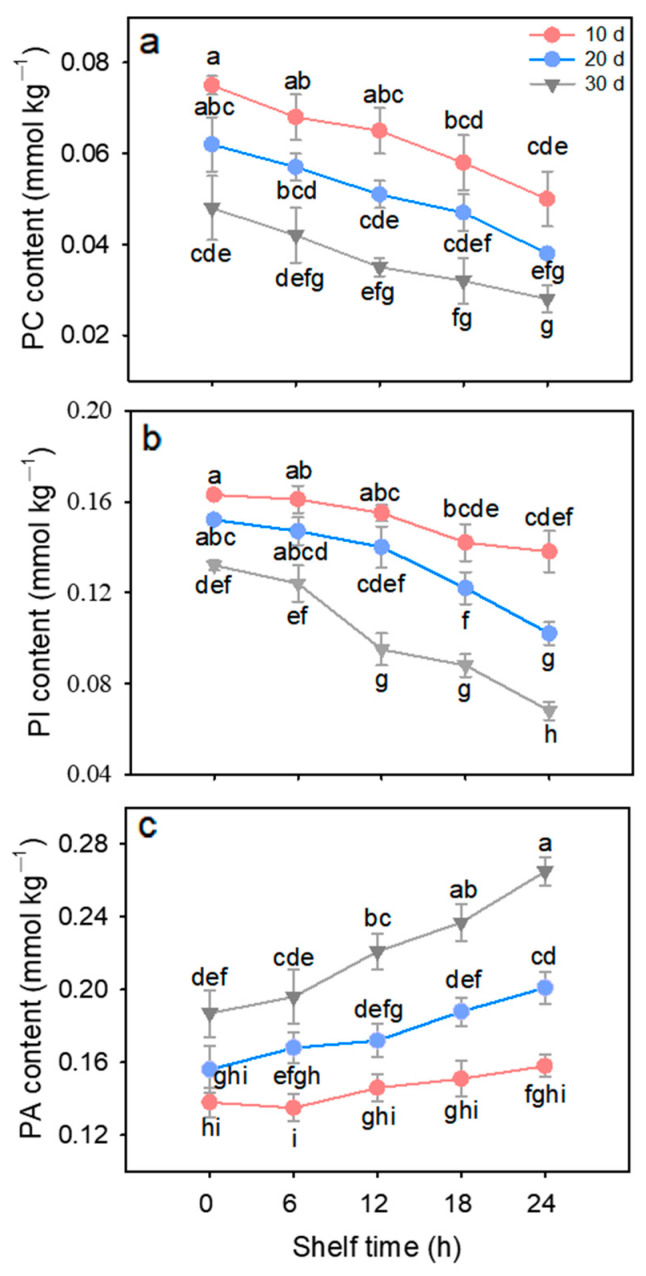
Changes in phosphatidylcholine (PC) (**a**), phosphatidylinositol (PI) (**b**), and phosphatidic acid (PA) (**c**) content of litchi fruits stored for 10, 20, and 30 days at 4 °C during subsequent 24 h shelf time at 25 °C. Data are shown as means ± SE (*n* = 3). Lowercase letters denote statistically significant differences at *p* < 0.05.

As shown in Figure 4a, after storage at 4 °C for 20 and 30 d, the PC content decreased by 18.4% and 35.6%, respectively, compared to the value of 10 d. Upon removal from low temperature, the PC content also decreased continuously with increasing shelf time. In addition, the longer the cold storage time, the faster the PC content decreased with the extension of the ambient shelf life. After storage at 25 °C for 24 h, the PC content of litchi fruits with 10, 20, and 30 d of cold storage decreased by 33.3%, 37.8% and 42.1%, respectively. The changes in PI content of cold-stored litchi fruits were similar to those of PC, both of which decreased significantly (*p* < 0.05) with the extension of cold and ambient storage (Figure 4b). The PI content of litchi fruits after 10, 20, and 30 d of cold storage decreased by 15.3%, 32.9%, and 48.5%, respectively. However, the PA content of litchi fruits increased significantly (*p* < 0.05) with prolonged cold storage duration (Figure 4c). After 10, 20, and 30 d of cold storage, the PA content of litchi fruits increased by 14.5%, 28.8% and 41.5%, respectively.

### 3.4. Contents of Anthocyanins, Total Phenolics, and Activities of Anthocyanase, PPO, POD, and PAL

**Figure 5:**
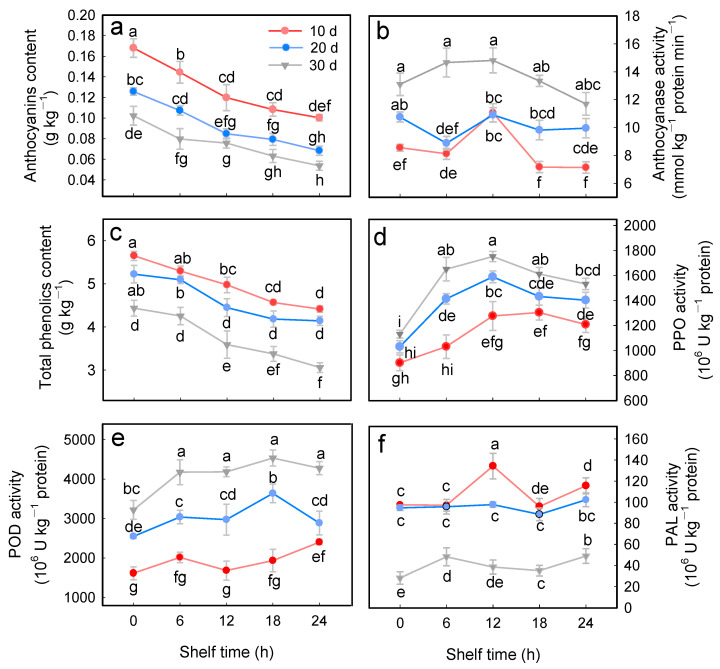
Changes in anthocyanins (**a**), anthocyanase (**b**), phenolic substances (**c**), PPO (**d**), POD (**e**), and PAL (**f**) activities of cold-stored litchi fruits stored for 10, 20, and 30 days at 4 °C during subsequent 24 h shelf time at 25 °C. Data are shown as means ± SE (*n* = 3). Lowercase letters denote statistically significant differences at *p* < 0.05.

The content of anthocyanins and total phenolics in the cold-stored litchi fruits decreased significantly (*p* < 0.05) with the extension of ambient storage time (Figure 5a,c). After storage at 4 °C for 30 d, the anthocyanins and total phenolics content decreased by 41.2% and 21.9%, respectively, compared to the fruits stored for 10 d. Moreover, after storage at ambient temperature for 24 h, litchi fruits stored at 4 °C for 20 and 30 d decreased the anthocyanin content by 26% and 50%, respectively. Meanwhile, the total phenolics content by 5.0% and 21.1%, respectively, compared to those 10 d of storage. This suggested that the longer the fruit is stored at low temperature, the faster the content of anthocyanins and total phenolics decreases.

The PPO activity of cold-stored litchi fruits rose and reached a peak value at 12 h during the shelf life at room temperature. The PPO activity in fruits stored for 30 d was approximately 37% and 11% higher than that in the 10 d and 20 d groups, respectively (Figure 5d). POD activity in cold-stored litchi fruits also exhibited an increasing trend depending on the storage duration. Specifically, the activity was highest in fruits stored for 30 d (4278.2 × 10^6^ U kg^−1^), which was approximately 1.5-fold and 3.6-fold higher than that in fruits stored for 20 d and 10 d, respectively (Figure 5e). Concurrently, after storing at 25 °C for 24 h, it was observed that the PAL activity of fruits stored in 4 °C for 30 d exhibited a 56.9% decrease compared to the fruits with 10 d of storage (Figure 5f). The anthocyanase activity in the litchi fruits after 30 d of cold storage reached 11.69 mM kg^−1^ protein, which was higher than the activity levels observed in fruits stored for 10 and 20 d, at 7.13 and 9.95 × 10^6^ U kg^−1^, respectively (Figure 5b).

### 3.5. Principal Component Analysis

**Figure 6:**
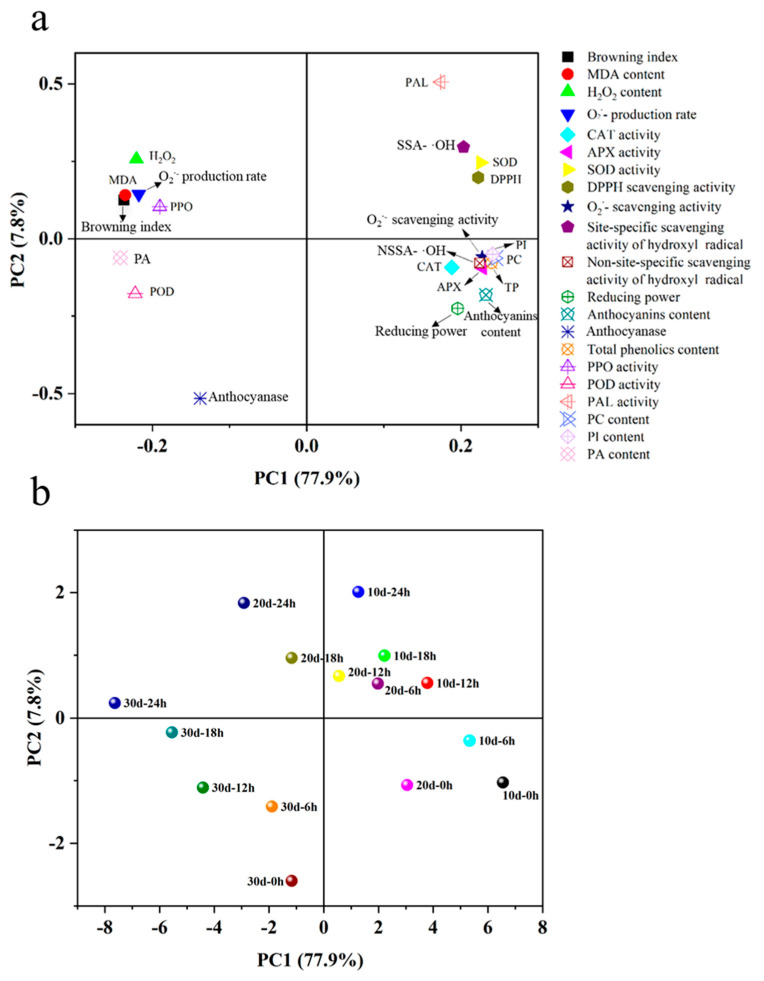
Principal component analysis of indicators of cold-stored litchi fruits stored for 10, 20, and 30 days at 4 °C during subsequent 24 h shelf time at 25 °C. Factor loading plot (**a**) and factor score plot (**b**). Abbreviations in plot A: SSA-·OH, site-specific scavenging activity of hydroxyl radical; NSSA-·OH, non-site-specific scavenging activity of hydroxyl radical; TP, total phenolic content; DPPH, DPPH scavenging activity. Data points in plot B are labeled as “xd-yh”, denoting first 4°C cold storage for x days, then ambient storage for y hours.

The PCA loading plot provides insights into the correlation between various parameters and the principal components (Figure 6a). As shown, the cumulative contribution of PC1 and PC2 accounts for 85.7% of the total variance, with PC1 contributing 77.9% and PC2 contributing 7.8%. This indicates that PC1 and PC2 together capture the majority of the sample information. From the plot, it is evident that indicators such as activities of PAL, CAT, APX, and SOD, site-specific scavenging activity of hydroxyl radicals, DPPH scavenging activity, PI, PC, reducing power, and anthocyanin content are positively correlated with PC1. In contrast, H_2_O_2_, MDA, browning index, PI, POD, and anthocyanase activity are negatively correlated with PC1. These correlations are consistent with the relationship between the quality and these indicators of litchi fruit. As shown in the score plot (Figure 6b), the 10 d and 20 d samples are closely clustered, while the 30 d sample is distinctly separated, indicating significant changes in the indicator characteristics of the 30 d sample. The samples at 10 d-0 h, 10 d-6 h, and 20 d-0 h are positioned closest to each other. Furthermore, another cluster is formed by the samples at 10 d-12 h, 10 d-18 h, 20 d-6 h, and 20 d-12 h.

## 4. Discussion

Cold chain logistics are vital for preserving perishable fruit and vegetables during transit. It reduces respiratory rates, mitigates physical damage and microbial growth, and maintains quality and sensory traits [23]. Advances in refrigeration and logistics enable efficient global distribution of perishable agro-products [23]. However, subsequent transfer to ambient temperature storage phases subjects cold-stored produce to accelerated deterioration in quality [3,24]. In this study, cold-stored litchi fruits suffered rapid pericarp browning after removal from 4 °C. Specifically, fruits stored for 30 d exhibited more severe browning during ambient shelf life than those stored for 10 and 20 d (Figure 2a). Under senescence or abiotic stress conditions, the dynamic balance between ROS generation and elimination would be disrupted, leading to excessive accumulation of ROS [25,26]. H_2_O_2_ is one of the most abundant ROS in cells, whose accumulation level can reflect the oxidation status and senescence progression [27]. MDA, a lipid peroxidation product, whose concentration quantitatively correlates with the cellular damage, thereby serving as a critical indicator for assessing senescence or stress responses [28]. As demonstrated in Figure 2b,c, the MDA content and H_2_O_2_ content in fruits stored for 30 d were higher than those in fruits stored for 10 and 20 d, and rapidly increased as shelf time at 25 °C progressed. These results revealed that a more pronounced senescence and quality deterioration occurred with the duration of cold storage.

To deal with the excessive accumulation of ROS and prevent oxidative damage, plants have evolved several enzymes that eliminate ROS, such as SOD, CAT, and APX [29,30]. Within this investigation, the activities of these three enzymes in the pericarp of cold-stored litchi fruits decreased with time at 4 °C. After transferring to 25 °C from 4 °C, the activities of these enzymes showed a rapid decrease during the ambient shelf time (Figure 3a–c). Generally, the enzymatic activities of SOD, CAT, and APX in litchi fruits stored for 30 d were lower than those in fruits stored for 10 and 20 d after cold storage and throughout ambient shelf time. Apart from the antioxidant enzyme system, plants also feature a non-enzymatic antioxidant system, which comprises low-molecular-weight antioxidants such as ascorbates and phenolics [27]. The evaluation of non-enzymatic antioxidant activity typically involves the assessment of reducing power and free radical scavenging activity [27]. In this present study, as storage time at 4 °C and shelf time at 25 °C progressed, the free radical (DPPH, ·OH, and O_2_^·−^) scavenging activity (Figure 3d–g) and reducing power (Figure 3h) declined. Moreover, fruits kept for 30 d had much less reducing power and free radical scavenging activity than fruits kept for 10 and 20 d during the shelf life. These findings suggest that extended cold storage can cause a reduction in activities of antioxidant enzymes after transferring to ambient shelf life, thereby impairing ROS scavenging efficiency and leading to the accumulation of ROS.

Excessive ROS accumulation subsequently triggered membrane lipid peroxidation. In plants, phospholipids, primarily displayed as phosphoglycerides (such as PC, PA, and PI), represent the majority of membrane lipids [31]. It has been demonstrated that PLD, as a phosphohydrolase, was capable of hydrolyzing the ester bond of PC to generate PA [31]. Our previous studies have demonstrated that prolonged cold storage of litchi fruit resulted in an increase in PLD activity [6]. PI can be hydrolyzed by phospholipase C (PLC) to generate the inositol-1,4,5-triphosphate (IP3), which can trigger protein kinase C and the intracellular calcium signal during stress response [32]. In the present research, the content of PC and PI in cold-stored litchi fruits exhibited a steady decline, while the content of PA showed an increase over time during the shelf life at 25 °C (Figure 4). Moreover, the alterations in the PC, PA, and PI content of litchi fruits are more pronounced after 20 and 30 d of cold storage than after 10 d. Therefore, it is reasonable to speculate that following the transition to 25 °C, the rise in activities of PLD and PLC occurs in tandem with the increase in temperature, thereby accelerating the degradation of PC and PI and the accumulation of PA, and ultimately leads to the destructive process of the endomembrane system.

**Figure 7:**
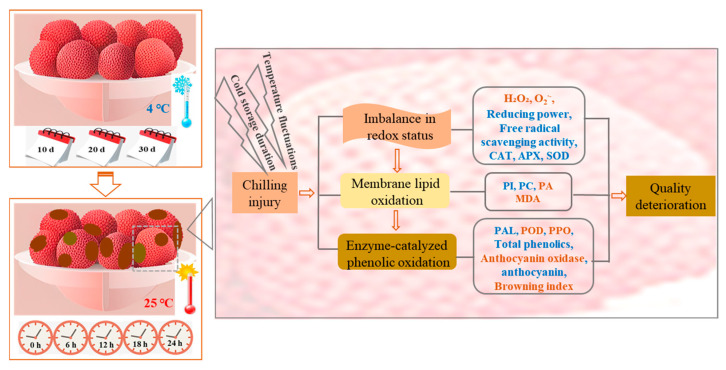
Possible mechanism of the effect of cold storage duration on the quality of litchi fruit during ambient shelf time. Red font indicates an increase in physiological indicators, while blue font denotes a decrease.

The browning of litchi is directly associated with the degradation of anthocyanins and phenolic compounds, an enzyme-catalyzed oxidation reaction driven by PPO and POD [9]. During senescence, the collapse of the endomembrane system allowed the phenolic substances to contact PPO and POD directly, thereby initiating the enzymatic reaction process [11,12]. The results in the present study also showed that activities of PPO and POD of cold-stored litchi fruits exhibited an increasing trend after transferring to ambient shelf life, and the litchi fruits stored at 4 °C for 30 d exhibited the highest activity (Figure 5d,e), which was aligned with the observed trend in the degradation of total phenolics (Figure 5c). The accumulation of anthocyanin is dependent upon the balance between biosynthesis and degradation [5]. The biosynthesis of anthocyanins starts with the phenylpropanoid pathway, in which PAL catalyzes the first step [33]. Previous reports have revealed that a decline in PAL activity may lead to the loss of anthocyanins in litchi fruit [5,34]. In this study, a reduction in PAL activity caused by prolonged cold storage time was observed (Figure 5f), which led to reduced synthesis of anthocyanins (Figure 5a) and total phenolics (Figure 5c). Anthocyanase catalyzes the hydrolysis of anthocyanins, whereby the β-1-4-glycosidic bonds of anthocyanins undergo cleavage to yield aglycone and free sugar [35]. Our result showed that the anthocyanase activity increased with extension of cold storage time (Figure 5b), which also contributed to the decrease in the content of anthocyanins (Figure 5a). Taken together with the above results, a possible mechanism of the effect of cold storage duration on the quality of litchi fruit during ambient shelf time was proposed (Figure 7).

Based on the physiological indicators evaluated in this study, a PCA plot was constructed (Figure 6). The results show that activities of PAL, CAT, APX, and SOD, site-specific scavenging activity of hydroxyl radicals, DPPH scavenging activity, PI, PC, reducing power, and anthocyanin content contribute to the quality maintenance of litchi fruit. Furthermore, the samples clustering indicates that to maintain the optimal quality of litchi fruit, it is recommended that the maximum storage duration at 4 °C should not exceed 10 d, followed by no more than 6 h at 25 °C before consumption. After 20 days of storage at 4 °C, followed by storing at 25 °C for a maximum of 12 h, the critical threshold for maintaining acceptable quality is reached. Exceeding storage durations results in a decline in litchi quality, which is no longer deemed acceptable for consumption. This pattern of temperature- and duration-dependent quality degradation is more sensitive than that observed in other tropical and subtropical fruits. For instance, mangoes and peaches exhibited deteriorations in nutritional quality and flavor compounds after storage at 5 °C for 25 d, followed by transfer to 25 °C for 3 d [36,37]. Similarly, jujube fruits showed pronounced quality deterioration after 35 d at 0 °C and subsequent shelf storage at 20 °C for 4 d [38]. This phenomenon is associated with the distinctive pericarp structure of litchi fruit, which is characterized by a thin cuticle and an underdeveloped wax layer that inadequately restricts water loss and buffers against external environmental fluctuations [39].

## 5. Conclusions

This study investigated the regulatory mechanisms and critical thresholds by which cold storage duration affects the quality deterioration of litchi fruit during ambient shelf life. Through an integrated analysis of physiological indicators related to oxidative stress, enzymatic browning, and membrane lipid metabolism, the critical interaction between cold storage duration and ambient exposure time was elucidated. This finding represents a significant advance beyond previous studies that focused solely on a fixed storage period. Furthermore, practical, time-dependent quality thresholds were identified by PCA, defining the optimal and critical limits for combined cold storage and ambient exposure. Our study not only advances the theoretical understanding of litchi postharvest biology but also provides a basis for optimizing preservation processes and reducing postharvest losses.

## Data Availability

The original contributions presented in the study are included in the article/Supplementary Materials; further inquiries can be directed to the corresponding author.

## Supplementary Materials

The following supporting information can be downloaded at: https://www.mdpi.com/article/10.3390/foods15010176/s1. Table S1: Pericarp water content of litchi fruits under different 4 °C cold storage durations and subsequent ambient shelf life conditions. All fresh weight (FW)-based indices in the present study can be converted to dry-weight (DW)-based values using the measured water content data. The conversion formula is as follows: DW-based content=FW-based content/(1-Water content). Figure S1: Litchi fruits harvested from the commercial orchard (a) and litchi fruits at 0 d (before cold storage, (b)). All treatment groups (cold stored for 10/20/30 d) used the same raw materials prior to storage.

## Abbreviations

The following abbreviations are used in this manuscript:

PCA	Principal component analysis
SOD	Superoxide dismutase
CAT	Catalase
PPO	Polyphenol oxidase
POD	Peroxidase
PAL	Phenylalanine ammonia lyase
ROS	Reactive oxygen species
PI	Phosphatidylinositol
PC	Phosphatidylcholine
PA	Phosphatidic acid
MDA	Malondialdehyde
PLC	Phospholipase C
IP3	Inositol-1,4,5-triphosphate
APX	Ascorbate peroxidase

## References

[1] Zhao L., Wang K., Wang K., Zhu J., Hu Z. (2020). Nutrient components, health benefits, and safety of litchi (*Litchi chinensis* Sonn.): A review. Compr. Rev. Food Sci. Food Saf..

[2] Liang H., Zhu Y., Li Z., Jiang Y., Duan X., Jiang G. (2025). Phytosulfokine treatment delays browning of litchi pericarps during storage at room temperature. Postharvest Biol. Technol..

[3] Liu H., Song L., You Y., Li Y., Duan X., Jiang Y., Joyce D.C., Ashraf M., Lu W. (2011). Cold storage duration affects litchi fruit quality, membrane permeability, enzyme activities and energy charge during shelf time at ambient temperature. Postharvest Biol. Technol..

[4] Yun Z., Qu H., Wang H., Zhu F., Zhang Z., Duan X., Yang B., Cheng Y., Jiang Y. (2016). Comparative transcriptome and metabolome provides new insights into the regulatory mechanisms of accelerated senescence in litchi fruit after cold storage. Sci. Rep..

[5] Mphahlele R.R., Caleb O.J., Ngcobo M.E.K. (2020). Effects of packaging and duration on quality of minimally processed and unpitted litchi cv. ‘Mauritius’ under low storage temperature. Heliyon.

[6] Park H., Eo H., Kim C., Stewart J., Lee U., Lee J. (2024). Physiological disorders in cold-stored ‘Autumn Sense’ hardy kiwifruit depend on the storage temperature and the modulation of targeted metabolites. Food Chem..

[7] Huang H., Liu H., Yu J. (2025). Delay of pericarp browning by chondroitin sulfate attributes of enhancing antioxidant activity and membrane integrity in litchi fruit after harvest. Postharvest Biol. Technol..

[8] Duan X., Liu T., Zhang D., Su X., Lin H., Jiang Y. (2011). Effect of pure oxygen atmosphere on antioxidant enzyme and antioxidant activity of harvested litchi fruit during storage. Food Res. Int..

[9] Wang C., Zhang S., Zhang D., Li F., Xie L., Dai T., Jiang Y. (2025). Gallic acid reduces pericarp browning of litchi fruit during storage. Postharvest Biol. Technol..

[10] Li T., Shi D., Wu Q., Zhang Z., Qu H., Jiang Y. (2019). Sodium para-aminosalicylate delays pericarp browning of litchi fruit by inhibiting ROS-mediated senescence during postharvest storage. Food Chem..

[11] Yu L., Zhou C., Fan J., Shanklin J., Xu C. (2021). Mechanisms and functions of membrane lipid remodeling in plants. Plant J..

[12] Liu H., Li J., Jiang Y., Li F. (2024). Identification and stability evaluation of polyphenol oxidase substrates of pineapple fruit. Food Chem..

[13] Roy Choudhury S., Pandey S. (2017). Phosphatidic acid binding inhibits RGS1 activity to affect specific signaling pathways in Arabidopsis. Plant J..

[14] Li F., Xu Z., Song L., Li L., Liu H., Liao Y., Zhao Y. (2026). Inhibition of postharvest soft rot in Chinese flowering cabbage by propyl gallate. Postharvest Biol. Technol..

[15] Liu G., Liu S., Liu J., Xiang Y., Zhu L., Xu X., Zhang Z. (2025). Trehalose delays postharvest browning of litchi fruit by regulating antioxidant capacity, anthocyanin synthesis and energy status. Postharvest Biol. Technol..

[16] Rahman M., Asaeda T., Fukahori K., Imamura F., Nohara A., Matsubayashi M. (2023). Hydrogen peroxide measurement can be used to monitor plant oxidative stress rapidly using modified ferrous oxidation xylenol orange and titanium sulfate assay correlation. Int. J. Plant Biol..

[17] Pang L., Jiang Y., Chen L., Shao C., Li L., Wang X., Li X., Pan Y. (2024). Combination of sodium nitroprusside and controlled atmosphere maintains postharvest quality of chestnuts through enhancement of antioxidant capacity. Foods.

[18] Hou Y., Gou M., Zhang Z., Meng L., Zhu L., Xu X., Li T., Jiang Y., Xiao J., Yang J. (2025). Application of Ferulic Acid to Alleviate Chilling Injury of Banana Fruit by Regulating Redox, Phenylpropanoid, and Energy Metabolism. Food Front..

[19] Zhang Y., Huber D.J., Hu M., Jiang G., Gao Z., Xu X., Jiang Y., Zhang Z. (2018). Delay of postharvest browning in litchi fruit by melatonin via the enhancing of antioxidative processes and oxidation repair. J. Agric. Food Chem..

[20] Xu D., Xi P., Lin Z., Huang J., Lu S., Jiang Z., Qiao F. (2021). Efficacy and potential mechanisms of benzothiadiazole inhibition on postharvest litchi downy blight. Postharvest Biol. Technol..

[21] Zhang Z., Pang X., Ji Z., Jiang Y. (2001). Role of anthocyanin degradation in litchi pericarp browning. Food Chem..

[22] Zhang S., Lin Y., Lin H., Lin Y., Chen Y., Wang H., Shi J., Lin Y. (2018). *Lasiodiplodia theobromae* (Pat.) Griff. & Maubl.-induced disease development and pericarp browning of harvested longan fruit in association with membrane lipids metabolism. Food Chem..

[23] Han J.-W., Zuo M., Zhu W.-Y., Zuo J.-H., Lü E.-L., Yang X.-T. (2021). A comprehensive review of cold chain logistics for fresh agricultural products: Current status, challenges, and future trends. Trends Food Sci. Tech..

[24] Liu D., Xu C., Guo C., Zhang X. (2020). Sub-zero temperature preservation of fruits and vegetables: A review. J. Food Eng..

[25] Liu J., Bao Y., Meng L., Xu X., Zhu L., Zhang Z. (2024). Physiological and transcriptomic analyses provide new insights into the regulatory mechanisms of accelerated senescence of litchi fruit in response to strigolactones. Food Biosci..

[26] Rudenko N.N., Vetoshkina D.V., Marenkova T.V., Borisova-Mubarakshina M.M. (2023). Antioxidants of non-enzymatic nature: Their function in higher plant cells and the ways of boosting their biosynthesis. Antioxidants.

[27] Zhang S., Shan Y., Li Y., Zhang J., He J., Qu H., Duan X., Jiang Y. (2024). Role of hydrogen peroxide receptors in endocarp browning of postharvest longan fruit. Food Front..

[28] Gürbüz G., Heinonen M. (2015). LC–MS investigations on interactions between isolated β-lactoglobulin peptides and lipid oxidation product malondialdehyde. Food Chem..

[29] Kapoor D., Singh S., Kumar V., Romero R., Prasad R., Singh J. (2019). Antioxidant enzymes regulation in plants in reference to reactive oxygen species (ROS) and reactive nitrogen species (RNS). Plant Gene.

[30] Wang P., Liu W., Han C., Wang S., Bai M., Song C. (2024). Reactive oxygen species: Multidimensional regulators of plant adaptation to abiotic stress and development. J. Integr. Plant Biol..

[31] Huang S., Bi Y., Li H., Liu C., Wang X., Wang X., Lei Y., Zhang Q., Wang J. (2023). Reduction of Membrane Lipid Metabolism in Postharvest Hami Melon Fruits by n-Butanol to Mitigate Chilling Injury and the Cloning of Phospholipase D-β Gene. Foods.

[32] Singh A., Bhatnagar N., Pandey A., Pandey G.K. (2015). Plant phospholipase C family: Regulation and functional role in lipid signaling. Cell Calcium.

[33] Yücetepe M., Özaslan Z.T., Karakuş M.Ş., Akalan M., Karaaslan A., Karaaslan M., Başyiğit B. (2024). Unveiling the multifaceted world of anthocyanins: Biosynthesis pathway, natural sources, extraction methods, copigmentation, encapsulation techniques, and future food applications. Food Res. Int..

[34] He M., Yin F., Dek M.S.P., Liao L., Liu Y., Liang Y., Cai W., Huang L., Shuai L. (2025). Methyl jasmonate delays the browning of litchi pericarp by activating the phenylpropanoid metabolism during cold storage. Postharvest Biol. Technol..

[35] Araya V., Gatica M., Uribe E., Román J. (2024). In silico analysis of the mecular interaction between anthocyanase, peroxidase and polyphenol oxidase with anthocyanins found in cranberries. Int. J. Mol. Sci..

[36] Zhang Z., Zhu Q., Hu M., Gao Z., An F., Li M., Jiang Y. (2017). Low-temperature conditioning induces chilling tolerance in stored mango fruit. Food Chem..

[37] Islam A., Acıkalın R., Ozturk B., Aglar E., Kaiser C. (2022). Combined effects of Aloe vera gel and modified atmosphere packaging treatments on fruit quality traits and bioactive compounds of jujube (*Ziziphus jujuba* Mill.) fruit during cold storage and shelf life. Postharvest Biol. Technol..

[38] Zhou H., Ye Z., Wang L., Zhang S., Yuan Z., Su M., Zhang X., Du J., Li X., Zhang M. (2025). 1-MCP regulates taste development in cold-stored peach fruit through modulation of sugar, organic acid, and polyphenolic metabolism. Postharvest Biol. Technol..

[39] Huang H., Liu H., Wang L., Xiang X. (2023). Cuticular wax metabolism responses to atmospheric water stress on the exocarp surface of litchi fruit after harvest. Food Chem..

