# Supplementary material for: Mechanisms and Critical Thresholds of Cold Storage Duration-Modulated Postharvest Quality Deterioration in Litchi Fruit During Ambient Shelf Life
Source: Foods. 2026 Jan 5;15(1):176. doi: 10.3390/foods15010176 (PMC12785488; doi:10.3390/foods15010176)
Supplement: Supplementary file 1 [file foods-15-00176-s001.zip › foods-4050725-supplementary.pdf]

Table S1. Pericarp water content of litchi fruits under different 4 °C cold storage durations and subsequent ambient shelf life conditions. All fresh weight (FW)-based indices in the present study can be converted to dry weight (DW)-based values using the measured water content data. The conversion formula is as follows: DW-based content=FW-based content/(1-Water content).

4 °C storage duration (d)	Ambient shelf storage time (h)	Water content (%)
10	0	71.5%
	6	70.9%
	12	70.2%
	18	69.0%
	24	68.5%
20	0	70.1%
	6	69.0%
	12	68.0%
	18	67.3%
	24	66.5%
30	0	68.2%
	6	67.3%
	12	66.2%
	18	65.1%
	24	64.0%

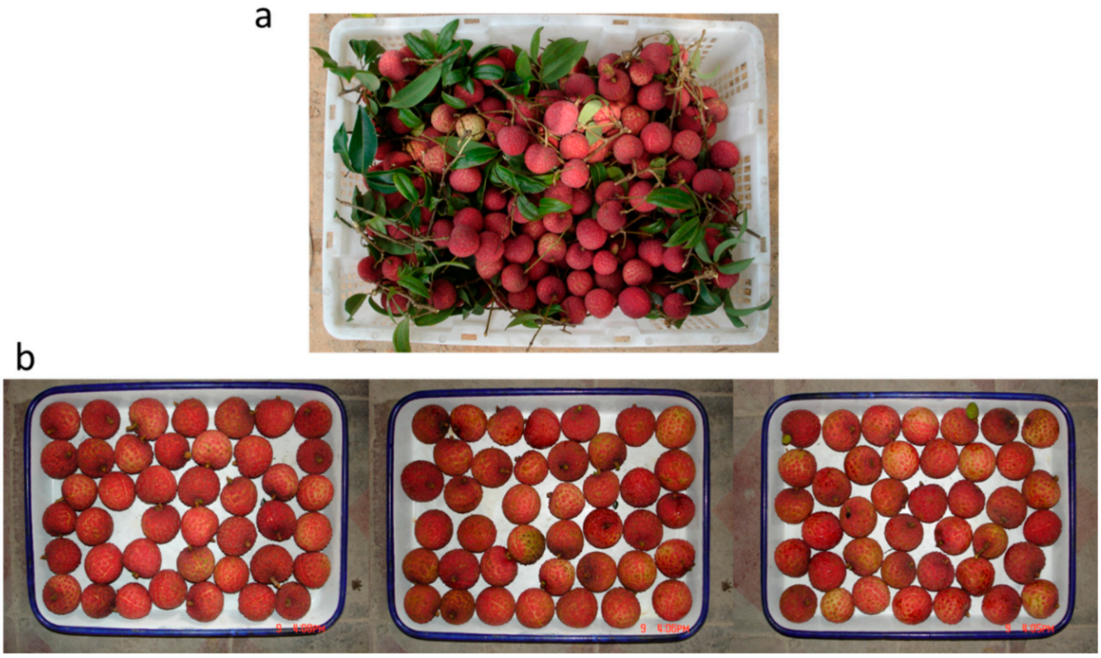

Figure S1. Litchi fruits harvested from the commercial orchard (a) and litchi fruits at 0 d (before cold storage, (b)). All treatment groups (cold stored for 10/20/30 d) used the same raw materials prior to storage.
